# Assessing the Contribution of Data Mining Methods to Avoid Aircraft Run-Off from the Runway to Increase the Safety and Reduce the Negative Environmental Impacts

**DOI:** 10.3390/ijerph17030796

**Published:** 2020-01-28

**Authors:** Olga Vorobyeva, Juraj Bartok, Peter Šišan, Pavol Nechaj, Martin Gera, Miroslav Kelemen, Volodymyr Polishchuk, Ladislav Gaál

**Affiliations:** 1 MicroStep-MIS, Čavojského 1, 841 04 Bratislava, Slovakialadislav.gaal@microstep-mis.com (L.G.); 2 Department of Astronomy, Physics of the Earth, and Meteorology, Comenius University in Bratislava, Mlynská dolina 4, 842 48 Bratislava, Slovakia; 3 Faculty of Aeronautics, Technical University of Košice, Rampová 7, 041 21 Košice, Slovakia; 4 Faculty of Information Technologies, Uzhhorod National University, Narodna Square, 3, 88000 Uzhhorod, Ukraine

**Keywords:** SESAR, safety, runway excursion, runway surface condition, data mining methods

## Abstract

The Single Europe Sky Air Traffic Management Research (SESAR) program develops and implements innovative technological and operational solutions to modernize European air traffic management and to eliminate the negative environmental impacts of aviation activity. This article presents our developments within the SESAR Solution “Safety Support Tools for Avoiding Runway Excursions”. This SESAR Solution aims to mitigate the risk of runway excursion, to optimize airport operation management by decreasing the number of runway inspections, to make chemical treatment effective with respect to the environment, and to increase resilience, efficiency and safety in adverse weather situations. The proposed approach is based on the enhancement of runway surface condition awareness by integrating data from various sources. Dangerous windy conditions based on Lidar measurements are also discussed as another relevant factor in relation to runway excursions. The paper aims to explore four different data mining methods to obtain runway conditions from the available input data sources, examines their performance and discusses their pros and cons in comparison with a rule-based algorithm approach. The output of the SESAR Solution is developed in compliance with the new Global Reporting Format of the International Civil Aviation Organization for runway condition description to be valid from 2020. This standard is expected to provide concerned stakeholders with more precise information to enhance flight safety and environmental protection.

## 1. Introduction

The European Air Traffic Management (ATM) system plays an important role in both European and global aviation, handling around 26,000 flights daily [1]. Nevertheless, it is based on aging technologies and practices that require fundamental modernization. For this very reason, the project Single European Sky ATM Research (SESAR) Joint Undertaking was launched in 2004. The SESAR project has a vital role in the research, development and implementation of innovative technological and operational solutions leading to increased ATM capacity, decreased delays and reduced cost and emissions [2].

One of the SESAR Solutions is Pj03b-06, named “Safety Support Tools for Avoiding Runway Excursions”. Excursions are among the most frequent runway safety accidents. According to the International Air Transport Association Safety Report, runway excursions caused 22% of all accidents over the 2010–2014 period [3]. The risk of a runway excursion is increased by wet and contaminated runways, in combination with gusts or strong cross/tail winds. The main goal of this SESAR Solution is to mitigate the risk of runway excursions by implementing on-board and ground systems and respective timely warnings. It should lead to significant benefits for ATM in terms of safety, efficiency and saving environment. This objective could be met by making a more elaborated usage of data from various sources like runway built-in sensors, meteorological sensors at airports, weather-based runway condition models, aircraft onboard sensors or surveillance radar data. This would result in a more precise and up-to-date information flow to flight crews. In addition, a 3D observation of the wind field in the vicinity of the airport using Lidar contributes to a complex assessment of runway excursion risk.

The new regulatory framework of the International Civil Aviation Organization (ICAO) requires Airport Operators (AOs) to report runway surface conditions to flight crews in a standardized manner (Global Reporting Format, GRF) in order to determine aircraft take-off and landing performance more accurately. AOs will have to assess the runway surface condition by using a Runway Condition Assessment Matrix (RCAM). As a result, AOs will assign a Runway Condition Code (RWYCC) to each third of each runway at the airport, based on the type, depth and coverage of water or contaminants. The established link between the runway surface conditions and the aircraft performance resolves the current problem of missing reference for friction measuring devices.

Our study attempts to address both the topic of the runway excursions and the implementation of the new reporting format in the framework of a novel approach, in order to increase the aviation safety and environmental protection. The new conceptual method involves the integration and processing of various input data sources, which results in an estimation of the RWYCC as a main output. One of the major novelties of our method is that the traditional approach of numerical modeling (represented herein by a rule-based algorithm) is replaced/supplemented by models based on data mining (DM) methods. We compared the performance of four different DM methods to calculate the RWYCC. 

Strong wind is another contributing factor to runway excursions along with the runway surface condition, represented by the RWYCC. Therefore, we also applied DM methods to handle the automated detection of wind shear based on similarities between the current Lidar 3D field of radial velocities and a conceptual model of microbursts. The motivation of this effort originated in the development of a fog prediction model based on DM [4], which has successfully been deployed in operative services as a part of the road monitoring and alerting system [5].

## 2. Materials and Methods 

### 2.1. Global Reporting Format

In 2008, the ICAO Friction Task Force commenced its work to address the shortfalls in the accuracy and timeliness of the assessment and reporting methods provided for in the present ICAO provisions and guidance material. Their goal was to develop provisions for the reporting of runway surface conditions and develop guidance on the operational requirements for airplane performance and for the assessment of runway surface conditions, including friction level. This resulted in the introduction of the new GRF transitioning from assessing the surface friction characteristics to runway surface condition assessment with consistent relation to aircraft braking performance [6]. 

A fundamental change in the GRF is the introduction of the RWYCC—a code number describing the runway surface condition—reflecting the runway braking capability as a function of the surface conditions. The RCAM (see Appendix A) is a basic tool for the RWYCC assessment from the observed runway surface conditions, also mapping the RWYCC to perceived braking action (BA) and lateral control of the aircraft during the landing roll. RWYCC and the type, the depth and the coverage of the runway contamination described by the RWYCC are consequently reported via the new comprehensive standardized Runway Condition Report (RCR) [6,7,8]. 

The concept of the RCR is premised on:

1) An agreed set of criteria used in a consistent manner for runway surface condition assessment, aeroplane (performance) certification and operational performance calculation;
2) a unique RWYCC, linking the agreed set of criteria with the aircraft landing and takeoff performance table, and related to the BA experienced and eventually reported by the flight crews;
3) reporting of the contaminant type and depth that is relevant to take-off performance;
4) a standardized common terminology and phraseology for the description of runway surface conditions that can be used by AO inspection personnel, air traffic controllers (ATCOs), aircraft operators and flight crew; and
5) globally harmonized procedures for the establishment of the RWYCC with a built-in flexibility to allow for local variations to match the specific weather, infrastructure and other particular conditions. [7]

The scope of the change induced by the implementation of the GRF is illustrated by the fact that at least ten ICAO documents have been amended in the discussed context: Annex 3, Annex 6, Annex 8, Annex 14 Volume I, Annex 15, Doc 9981, Doc 10066, Doc 4444, Doc 10064 and Doc 9137 [6].

### 2.2. SESAR Validation Process

The SESAR Solution may be divided into two distinctive parts: the airborne and the ground-based parts. The validation exercise related to the first, airborne part was conducted by Dassault with the support of the Direction des Services de la Navigation Aérienne (French Air Navigation Service Provider). Since one of the key topics of the current paper is related to the second, the ground-based part of the SESAR Solution, the next paragraphs will introduce it in more detail. 

The Runway Condition Awareness Management System (RCAMS), developed within this research, supports the staff responsible for runway assessment and maintenance. Unlike the current method of discontinuous visual inspections of runway conditions, the RCAMS enhances the awareness of runway conditions in a non-disruptive and continuous (uninterrupted) manner. It contributes to an optimization of airport operation management by decreasing the number of runway inspections, supports decision making for effective chemical treatment and increases resilience and safety in adverse weather situation. 

The ground-based part of the SESAR Solution (i.e., the RCAMS) was validated by three different partners at three different sites:

- Polish Air Navigation Services Agency and Warsaw University at Gdansk Lech Walesa Airport,
- Air Navigation Services of the Slovak Republic and MicroStep-MIS at Poprad–Tatry Airport,
- Aéroports de Paris at Paris Charles de Gaulle Airport.

The RCAMS validation exercises were supplemented and enriched by an On-board Braking Action Computation System (OBACS) prototype developed and validated by Airbus. In order to cover all 13 validation objectives allocated to this SESAR Solution, a workshop was organized to enhance the AO awareness for cases when the runway surface condition presents a critical status, which may impact airport operations. Moreover, the workshop involved Airspace Users and ATCOs to define runway excursion risk alert mechanisms and the alert interoperability with flight crews’ alerts.

The RCAMS provides the automatic means for the AOs to continuously assess a RWYCC on each third of each runway of the airport. One RCAMS platform was installed and validated at Poprad–Tatry Airport, which is a regional single runway airport located in the north of Slovakia. Due to its location near to the highest mountains of Slovakia and elevation exceeding 700 m above the sea level, this airport provides a wide range of adverse runway conditions. The validation exercise of the RCAMS at Poprad–Tatry Airport addressed four system components:

1) *Sensors and data acquisition*: The system acquired the data necessary to run the calculation of the RWYCC. The available Computed BA reported by OBACS was used as a potential downgrade criterion for the calculated RWYCC.
2) *Processing algorithms*: The collected data were processed to calculate the RWYCC.
3) *Prediction*: The current runway surface condition and the output from both the runway surface condition model and the numerical weather prediction model were processed to calculate the predicted RWYCC.
4) *Interpretation of outputs*: Human–Machine Interfaces (HMIs) presented relevant information to stakeholders: AOs (enabling also manual intervention according to the results of visual inspections and maintenance procedures) and ATCOs (read-only display with final results).

As a main means of validation, the RWYCC computed by the system was compared to the manually recorded RWYCC value based on disruptive runway inspection and friction measurement summarized in the SNOWTAM message [9]. The contaminant type and the depth, as well as the coverage per each third of runway from SNOWTAM, were translated to the RWYCC in accordance with the RCAM (Appendix A). Another means of validation were questionnaires for concerned users, AOs and ATCOs.

### 2.3. Available Data Sources

This paper processes data from a SESAR validation exercise period from mid-February to April 2018, and also from the following winter season (November 2018–April 2019) at Poprad–Tatry Airport. The collected dataset consists of data of different origins, including data from Runway Surface Condition sensors, meteorological observations, and the previous SNOWTAMs containing information about the runway surface condition based on the manual inspection of the runway. Table 1 lists all parameters used for the RWYCC computation with their sources and time resolutions.

In order to compute the RWYCC using DM models, we derived the RWYCC as a target attribute from human observations in SNOWTAMs. The observed contaminant type and depth was translated in compliance with the GRF described in Section 2.1. The database for learning the DM models to calculate the RWYCC was finally comprised of the following parts: data from Runway Surface Condition sensors and Automated Weather Station (AWS) listed in Table 1, subsurface temperature and general meteorological data such as global radiation, wind speed and direction, air pressure and relative humidity.

### 2.4. Rule-Based Algorithm for the RWYCC Calculation

The basic tool for the implementation of the GRF is the RCAM (Appendix A). However, a full RCAM scope with all contaminant types (including those with two layers) and additional characteristic is currently not achievable by any technical system except for human observations, and thus cannot be applied directly onto input data. Therefore, we proposed a set of rules to proceed from the available input data (described in Section 2.3) to a calculation of the RWYCC following the RCAM logic. 

The baseline for the calculation of the RWYCC by a rule-based algorithm is built on the last available observation of the contaminant type and depth and the resulting RWYCC (an application of the RCAM on the observations). The reliability of this information decreases with time. Therefore, the persistence of the last observed runway condition (and the related RWYCC) is continuously crosschecked by data from the sensors. 

An important RCAMS component for runway condition monitoring is a pair of passive and active sensors embedded into the runway at each third of the runway. From passive sensors, the measurement of water film height is used as the first criterion to confirm the persistence of the last observed runway condition or to indicate the trend associated with the information regarding the sensor contaminant type. Since the runway built-in sensors provide only point measurements at three specific locations on the runway, they lack information about spatial variations. Moreover, sensor capabilities are limited from the aspect of full RCAM scope and reliability of measurements (especially sensor contaminant type).

Therefore, the next criterion for the confirmation of persistence or the indication of a change is the measurement of precipitation, since type and intensity of precipitation significantly impact the state of the runway surface. The precipitation indicator sensor provides a reliable binary parameter and if it indicates precipitation, its type (phase) and intensity are retrieved from a disdrometer. This information is further crosschecked by a rain gauge and METAR messages. A confrontation of the last observed contaminant type and the resulting RWYCC based on precipitation requires the assessment of freezing conditions. The current runway temperature and the current freezing point temperature, measured by the active runway sensor, are compared. This information also enables us to identify potential phase changes of contaminants (e.g., the melting of snow or freezing of water on the runway). High relative humidity (near 100%) indicates the possibility of dew or frost formation on a previously dry runway. Finally, the runway (or air) temperature distinguishes between RWYCC 3 and 4 for compacted snow on the runway (according to the RCAM). These additional criteria support the rule-based algorithm to identify and eliminate the obvious inconsistences between the sensor contaminant type and depth and the observed weather conditions at the airport (e.g., there is intense rain at the airport and runway sensors still reports a dry runway), contributing to situational awareness.

Considering the last observation crosschecked by all available and relevant inputs for a particular weather situation, the rule-based algorithm assigns some probability to each RWYCC from 6 to 0 (based on expert judgment). The final RWYCC (used for algorithm performance assessment) is then the one with the highest probability. The RWYCC is supplemented by information about a consistency check against the last manually observed contaminant type, depth and the associated RWYCC in order to support decision making for the runway inspection execution or the application of the mechanical/chemical treatment of the runway.

### 2.5. Data Mining Methods

Data mining (DM) is a general approach where extensive databases are analyzed with the goal of obtaining new and potentially useful information on the future behavior of the investigated phenomenon, which is also called the target event. Tan et al. [11] described neural networks and decision trees, which are typical examples for creating models within the DM process. The procedures of DM, as detailed in the CRISP-DM methodology, were used in our research. CRISP-DM represents an industry initiative to summarize the previous practical experience and to develop a standard for the entire DM process [12].

Data preparation and cleaning are important phases of the CRISP-DM methodology that impact the final success of the models. All measured values were subjected to quality checks (valid range of each parameter, temporal consistency assuring that the change between two successive values is realistic, and consistency between different physical parameters) before the learning process. Manually observed numerical values were checked against textual logs of the runway status. We identified very low amount (less than 0.1 %) of records with missing data, and these were excluded from further processing. Due to the negligibly low percentage of missing records, we didn’t expect any significant influence of the data exclusion on the final results.

In this paper, we applied supervised machine learning models implemented in the 64-bit edition of the open source programming language R, version 3.5.1 [13]. The models required previous knowledge of experiment realizations (e.g., assessment of runway surface conditions and measurements of meteorological conditions) and made predictions by learning decision rules utilizing the previous experience. The “target event” was the RWYCC (based on SNOWTAM messages) as the main parameter representing the runway surface condition awareness, which contributes to avoiding runway excursions. The rest of the analyzed database (predictors) consisted of data from Runway Surface Condition sensors and AWS (see also Section 2.3). We chose four machine learning methods for the RWYCC calculation:

- Classification tree
- Linear Discriminant Analysis (LDA)
- *K*-nearest Neighbors (KNN)
- Artificial Neural Network (ANN).

A classification tree is a set of computer-generated rules, usually based on comparing features with thresholds. It consists of nodes of two types: terminal and non-terminal. Each non-terminal node has exactly two descendants and one parent, except of the root node with no parents. Comparing the entropy of the parent node with the entropy of the descendant nodes, one obtains the information gain [11], or, in other words, the expected reduction of the entropy, caused by sorting the records into descendant nodes. Alternative algorithms use different splitting measures, e.g., the GINI index or variance [14], but these were not used within this work. Typically, the construction of a classification tree consists of creating a maximal tree and then pruning it to get the tree smaller. The biggest advantage of a constructed classification tree is that it can be easily understood by humans and simply implemented into systems [12,15]. We used the R library *rpart* [16] with the parameter *method* set to *class* and the *splitting index* set to *information*, which meant building of classification trees where the information gain served as the splitting measure.

LDA aims at creating a classification rule for resolving objects into *m* distinct categories. LDA assumes that feature vectors have a multivariate Gaussian distribution with the same non-singular covariance matrix. The training dataset consists of feature vectors $x_{k}\in \;R^{p}$, $k\;\in \;\left\{1,\;\ldots ,\;n\right\}$, where *n* is total number of feature vectors and known classifications $c_{k},\;k\;\in \;\left\{1,\;\ldots ,\;n\right\}$ and *p* is the dimension of feature vectors.:

(1) $$\pi_{k}\left(x\right)=\frac{\exp\left(-\frac{1}{2}{\left(x-\mu_{k}\right)}^{T}\sum^{-1}\left(x-\mu_{k}\right)\right)}{{\left(2\pi \right)}^{p/2}\sqrt{\det\left(\sum \right)}}$$

In (1) $\pi_{k}$ is the underlying probabilistic distribution and $\mu_{k}$ is the mean value of the Gaussian distribution, $k\;\in \;\left\{1,\;\ldots ,\;n\right\}$.

(2) $$\delta_{k}\left(x\right)=\;x^{T}\sum^{-1}\mu_{k}-\;\frac{1}{2}\mu_{k}^{T}\sum^{-1}\mu_{k}+\;\log\pi_{k},\;k\;\in \;\left\{1,\ldots ,m\right\}$$

Equation (2) is then the discriminant equation with matrix notation [15]. We utilized the R library *MASS* [17] with default settings. 

The KNN method is based on finding the *K* nearest classified feature vectors to the yet non-classified object. Then the object is classified according to the maximal occurrence of categories among the *K* nearest neighboring feature vectors. KNN is considered as one of the simplest machine learning methods [18,19]. The R library *class* [20] with the parameter *k* equal either to *1* or to *3*, respectively, helped us to create two versions of the KNN model.

The ANN method is inspired by biological neural networks. An ANN is like a group of connected nodes (neurons = nodes, connections = synapses) that can transmit signals. When the weighted sum of the input signals to the neuron is greater than the threshold, the neuron will fire and send a signal to a further neuron (node). The aim of ANN is to find weights and thresholds for each neuron (node) and its synapses (connections) [21,22]. We applied a single-hidden-layer feedforward neural network with 40 units in the hidden layer implemented in the R library *nnet* [23]. The amount of the hidden neurons (40) was estimated empirically after several experiments. 

### 2.6. Data Mining Method for Adverse Wind Conditions

When a wet/contaminated runway occurs simultaneously with a gusty wind, wind shear or strong tail/cross wind (all associated with microburst events), the risk of adverse effects to the plane’s braking performance increases. The wind field poses separate safety issues in itself; some major aircraft accidents have been caused by adverse wind conditions [24,25]. To incorporate wind information into the alerting system, we decided to use 3D Lidar information in addition to the standard measurements at an airport, which are performed at point locations only. Lidar is a remote sensing instrument for observing the properties of the atmosphere that are particularly related to the motion of the air and is capable of measuring the radial wind speed in an approx. 6 km radius around an airport [26]. 

Computationally, the detection/identification of microbursts is a complex problem. Because of the relatively short time that this phenomenon endures, in order to be handled by an alerting system, it must be recognized in its early phases. The detection of wind shear on the basis of radial velocities from Lidar is not straightforward—it requires trained personnel constantly monitoring the scans. In principle, it should be possible to automate this by means of pattern recognition (on the basis of divergence detection in the wind field, e.g., [27,28]) and train an ANN accordingly. Nonetheless, the natural domain of an ANN is real numbers, not the discrete elements, therefore, in essence, an ANN only calculates the probabilities of the presence of the desired pattern in a given location. If the estimated probability is high enough, an alarm shall be issued. In practice, however, the employment of ANN is not straightforward, since the microburst-induced wind shear situations occur very rarely. When we take into account the Lidar’s relatively limited (6 km) maximum range around an airport, the chance of detecting a microburst in the Middle European climate is very low. For example, during the time the Lidar has been operating at Bratislava Airport, we were able to capture only two occurrences of microbursts, which is not sufficient to train any ANN.

## 3. Results

### 3.1. The New Conceptual Method Developed Within SESAR

While the introduction of the new GRF (described in Section 2.1) resolves the problem of missing reference for friction measuring devices, the SESAR method aims to overcome the discontinuity of the runway surface condition data from visual inspections of the runway. The new concept of monitoring and reporting of the runway surface condition is based on the integration of various data sources. All these data assist to provide flight crew with the information on the runway surface condition in compliance with the new ICAO regulation (GRF) to increase safety and avoid runway excursions. Within the SESAR framework, the methodology of the RWYCC calculation and distribution was developed and is presented in Appendix B and Appendix C. It encompasses both the processes currently in use and the newly introduced ones.

As depicted in Appendix B, a system for the elaboration of the runway surface condition uses either runway built-in sensors and/or visual observations from the AO, weather data and forecast from meteorological services, flight crew report on experienced BA by transmitted BA (PIREP message) and/or broadcasted Computed BA (from OBACS), and optionally the braking performance of landed aircraft based on surveillance radar data. As a result the RWYCC is derived by a rule-based algorithm or using DM methods. The RCAMS allows for the continuous monitoring of the current runway surface condition in between two visual observations and eventually triggers a new visual inspection if a significant change is identified. The current runway condition awareness is supplemented by a forecast to elongate the usability of the runway condition status and hence improve planning. The RCAMS copes with this new conceptual scheme and so, aims at the mitigation of runway excursion risk.

The AO responsible for monitoring and reporting of the runway condition has the possibility to adjust the information to be disseminated according to his experience. The scheme of sharing and utilizing the information about the runway surface condition (contained in RCR) in Appendix C points out the information flows from the AO to all concerned stakeholders. A dedicated HMI has been developed to ensure the continuous monitoring of the runway condition and to enable the composing and dissemination of the RCR message. This HMI also facilitated the validation process of the declared RCAMS’s functionalities and improvements via a questionnaire. After the experience with the RCAMS using an HMI, the responsible airport staff had the chance to answer questions related to the fulfillment of validation objectives, perceived usefulness, ease of use and satisfaction with the system.

### 3.2. Data Mining Methods Scores

In the SESAR validation exercise, we implemented a rule-based algorithm for the RWYCC calculation (principal output of the RCAMS). The utilization of DM methods represents another approach to estimate the RWYCC from the available data sources. Instead of the direct application of the RCAM onto input data, we trained DM models using historical data sets containing available input sources from sensors. Human observations by professional AO staff served as the target attribute, as we assumed the correct application of the RCAM´s full scope. The subdivision of data into a training set and a test set that was excluded from the training process enabled the evaluation of the DM models. We applied four different DM methods, as described in Section 2.5. To quantify the performance of the individual DM methods on the RWYCC calculation, we have computed four different scores [29]:

- *Accuracy* (ACC), see Equation (3)
- *Heidke skill score* (HSS), see Equation (4)
- *Hanssen-Kuipers discriminant* (HK), sometimes also termed as Pierce skill score, see Equation (5)
- *Kuiper skill score* (KSS), see Equation (6).

These scores are derived from a multi-category contingency table (Table 2). In Table 2, *n(F_i_, O_j_)* corresponds to the number of forecast events in category *i* and the observed events in category *j*. The total number of the forecast events in category *i* equals *N(F_i_)* and the observed events in category *j* is *N(O_j_)*. *N* represents the overall sum of forecast or observed events. A perfect model would have non-zero values only at the diagonal of the table for *n(F_i_, O_j_), i = j*, otherwise *n(F_i_, O_j_) = 0* for *i ≠ j*. *K* is the number of categories. 

Following the convention used in Table 2, the four scores introduced above can be expressed using Equations (3)–(6):

(3) $$ACC=\;\frac{1}{N}\overset{K}{\underset{t=1}{\sum }}n\left(F_{t},O_{t}\right)$$

(4) $$HSS=\;\frac{\frac{1}{N}\sum_{t=1}^{K}n\left(F_{t},O_{t}\right)-\;\frac{1}{N^{2}}\sum_{t=1}^{K}N\left(F_{t}\right)N\left(O_{t}\right)}{1-\;\frac{1}{N^{2}}\sum_{t=1}^{K}N\left(F_{t}\right)N\left(O_{t}\right)}$$

(5) $$HK=\;\frac{\frac{1}{N}\sum_{t=1}^{K}n\left(F_{t},O_{t}\right)-\;\frac{1}{N^{2}}\sum_{t=1}^{K}N\left(F_{t}\right)N\left(O_{t}\right)}{1-\;\frac{1}{N^{2}}\sum_{t=1}^{K}N{\left(O_{t}\right)}^{2}}$$

(6) $$KSS=\;\frac{\frac{1}{N}\sum_{t=1}^{K}n\left(F_{t},O_{t}\right)-\;\frac{1}{N^{2}}\sum_{t=1}^{K}N\left(F_{t}\right)N\left(O_{t}\right)}{1-\;\frac{1}{N^{2}}\sum_{t=1}^{K}N{\left(F_{t}\right)}^{2}}$$

In the following five subsections (3.2.1‒3.2.5), we will present the most relevant statistics (mean, standard deviation (Sd), variability (Var), minimum (Min), maximum (Max), median, skewness and kurtosis) of the scores related to the individual DM methods. An inter-comparison of the individual methods (also in terms of box-plots) and their comparison with the performance of the Persistence model (assuming the persistence of the previous status) is introduced in Section 3.2.6. The ultimate discussion of the performance of the DM methods in the light of the rule-based algorithm can be found in Section 4.

#### 3.2.1. LDA Results

This subsection provides the statistics for the scores of the LDA method. The RWYCC, based on the SNOWTAM from Poprad–Tatry Airport, was split into a training set (approx. 60%) and a test set (40%). The multi-category contingency table contains mean values based on 1000 LDA models on test data. The statistics resulting from the scores for each of 1000 models using the LDA method are summarized in Table 3.

#### 3.2.2. KNN Results for K = 1

Table 4 contains the scores with their statistics based on 1000 models using the KNN method for the parameter *K* = 1. The training set comprised 60% of the available data set‒the rest were test data.

#### 3.2.3. KNN Results for K = 3

This subsection presents the scores for the same DM method (KNN), but considers three nearest neighbors (*K* = 3). The statistics for the scores (Table 5) are based on 1000 models while splitting the entire data into a training set (60%) and a test set (40%). 

#### 3.2.4. ANN Results

The outcomes for the ANN method are divided into two parts; the first group represents the scores for the training set, comprising 70% of the complete dataset (Table 6), whereas the second one is the same, but for the test set (Table 7). 

#### 3.2.5. Classification Tree Results

The statistics for the scores based on 1000 models using the Classification tree method with two thirds of the data as the training set and the remaining third as the test set are summed up in Table 8. 

#### 3.2.6. An Inter-Comparison of the DM Methods

**Figure 1:**
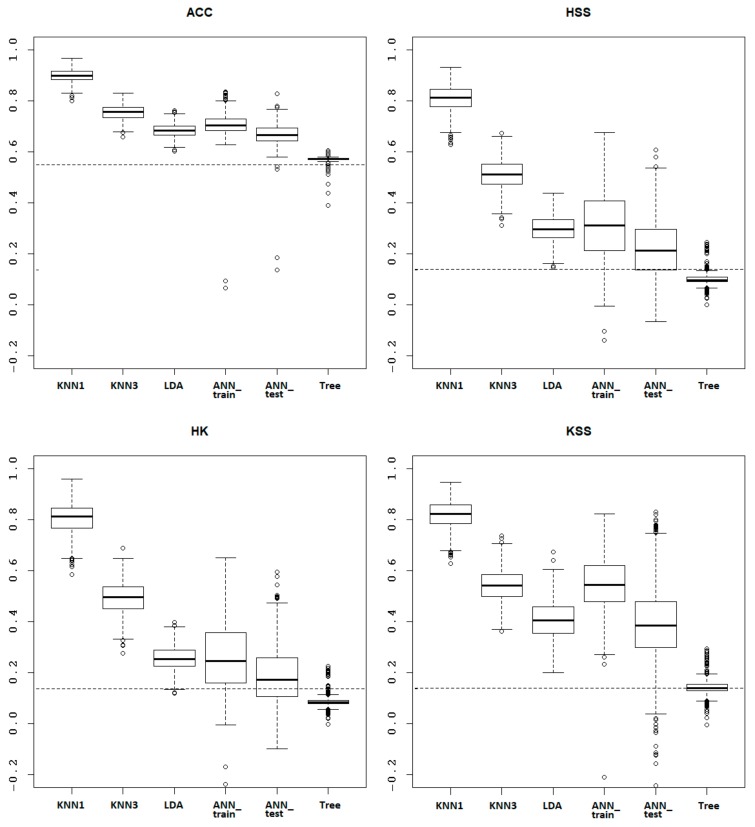
Performance scores (ACC, HSS, HK and KSS) for the DM methods KNN, *K* = 1 (KNN1), KNN, *K* = 3 (KNN3), LDA, ANN on training data set (ANN_train), ANN on test data set (ANN_test) and Classification tree (Tree). The dotted horizontal line represents the Persistence model. For further abbreviations, see Appendix E.

The current subsection discusses the performance of the individual DM methods in comparison to each other, and to the Persistence model, respectively. Figure 1 presents box plots of the score statistics based on the test data sets, with the DM algorithms sorted in a descending order according to the mean score values. The only exception is the ANN method, where the results related both to the training data set and the test data set are displayed next to each other (and hence, the box plot related to the ANN training data set slightly distorts the arrangement of the remaining box plots in the descending order). The similarities of these two ANN-based results indicate that the ANN is not overfitted. Once an ANN is overfitted, it loses the ability to generalize new data beyond the training set. In such cases, it would resemble the training data set instead of learning the inherent patterns of the data. 

All the adopted DM algorithms outperformed the Persistence model, except for the Classification tree. The best performing models were the two versions of the KNN method, probably thanks to the best resistance to unbalanced input data [30]. The order of the models according to the mean score values is identical for all performance scores with the ACC at the first position, and followed by the HSS, HK and KSS scores. Such a degree of consistency confirms the relative order of successfulness of the tested models when applied to the examined problem. 

### 3.3. Microburst Model for Data Minig Method

To solve the issue of the scarcity of the target attribute (Section 2.6), we created a microburst model: we generated a wind field corresponding to the Lidar scans with the presence of a simulated microburst. We positioned the microburst at different locations within the approx. 10 × 10 km domain centered at the airport and varied its parameters. In this approach, the training base for the ANN model consisted of a collection of simulated wind fields with microbursts. We used the individual Lidar beams (scanning the entire area and forming a 2D-raster) as the problem space. Thus, there were as many neurons in each input layer of the ANN as the number of gates along a Lidar beam. For each gate (i.e., neuron), we used the radial velocity measured by Lidar as an input value and let the ANN calculate the output value: the probability, that the respective point along the beam is involved in a microburst. The precious cases with real microbursts were used as testing sets for the ANN model verification. In both cases, the ANN identified the real microburst in the Lidar data. These results are promising; however, no statistically robust statements can be derived yet.

## 4. Discussion

A RWYCC is a principal characteristic informing pilots about the runway surface conditions. Once correctly assessed, the knowledge of the RWYCC mitigates runway excursion risk due to its correlation with the aircraft braking performance. Based on the results presented in Section 3, we can presume the application of DM methods to the estimation of the RWYCCs to be a promising approach. 

All investigated DM methods for the RWYCC calculation reached better scores in comparison with the accuracy (54.8%) of the Persistence model (assuming the persistence of the previous RWYCC based on manual observation). The KNN (*K* = 1) method achieved the best performance among the examined methods. Its mean accuracy of 90.1% and other scores with means over 0.8 based on 1000 models (with different splitting of the dataset into training and test data) makes this DM method the most successful and meaningful for the RWYCC calculation. This performance stems also from the resistance of the KNN (*K* = 1) method to unbalanced input data, since, for the categorization of new measurements, only information from the nearest neighbor is necessary. The mean accuracy of all other methods ranged from 65% to 75%, except for the Classification tree method, reaching the mean accuracy only slightly higher than the Persistence model (56.9%). This DM method is probably the most affected by an unequal distribution of the RWYCCs, and thus, is rather not suitable for our purposes. A complete evaluation of the performance of the examined DM methods and their comparison with the Persistence model is discussed in Section 3.2.6 and compiled into a graphical form in Figure 1 ibid. 

The utilization of all DM methods is limited by the low (or even zero) number of some categories (e.g., wet ice on the runway being RWYCC 0 according to Appendix A). It is caused by the rare occurrence of the triggering weather phenomena and also by the runway treatment policy, especially at busy airports (from this point of view, the selection of Poprad–Tatry Airport, with less intense traffic, seems to be a good choice of experimental site). This strongly emphasizes the need to continue in the research by collecting of further rarely occurring situations.

The low quantity of infrequent RWYCCs, on the other hand, is not a constraint for the rule-based algorithm representing a physical model built on the available runway surface condition and weather data. However, its scores did not reach the expected performance level compared to the DM approach, indicating the necessity of further development and improvement. One of the most striking deficiencies of the rule-based algorithm is that it is not very flexible in the implementation of changes induced by its operation. It cannot easily suppress the importance of unreliable input parameters. Runway built-in sensors provide information about the contaminant type and depth, which are crucial for the RWYCC computation using the RCAM. Therefore, they significantly contribute to the final output of the rule-based algorithm (see also Section 2.4). A wrong assessment of contaminant type or depth by the runway built-in sensors was, in the vast majority, the main factor of the incorrect output of the rule-based algorithm. The limitations of these sensors come from both natural reasons (point measurements only, which are insufficient in heterogeneous conditions, limited scope of contaminant type assessment considering the full RCAM´s scope) and the low reliability of the contaminant type assessment. The most recurring case was a dry condition being reported by sensors while the runway was covered by dry snow or frost. 

In contrast to this, DM methods are much more resilient regarding the reliability of input data. Moreover, their performance grows in time as the available database for data analysis enlarges. The DM models estimate RWYCC directly (not using the RCAM) from input parameters with appropriate weighting factors. This enables a DM model to assign low priority to unreliable parameters from the runway built-in sensors (see Appendix D). On the contrary, most of the commonly used DM methods require an almost balanced multiplicity of observed categories of the target event, which is difficult to achieve for rare phenomena. The designed system is useful for airport management to fulfill the tasks of improving safety and environmental management in practice. Additionally, DM methods can be successfully used for the detection and/or prediction of other safety-related weather phenomena, e.g., adverse wind conditions.

## 5. Conclusions

The demand for an increase in safety motivated ICAO to introduce a new GRF for the observation and reporting of runway surface conditions with a closer relation to aircraft performance than the current methods. Coping with the full RCAM´s scope requires the visual inspection of the runway, which is associated with runway closure. In order to optimize the number of runway inspections and to support decision making for runway maintenance and chemical treatment, the current paper examined different methods for enhanced automatic means of continuous assessment of the runway surface condition. This contributes to increasing flight safety and environmental protection.

We presented the core results of the SESAR project Pj03b-06 “Safety Support Tools for Avoiding Runway Excursions”, which defines a new conceptual method based on the integration of multiple data sources. The RCAMS developed and validated within this SESAR Solution represents such a decision support tool for the staff responsible for runway assessment and maintenance to improve awareness of runway condition. The HMI of the RCAMS displays all the necessary information, including the calculated RWYCC characterizing the runway surface condition. In order to obtain the RWYCC, we examined four different DM methods, from which the *K*‒nearest neighbor method with *K* = 1 showed the best scores, with mean accuracy of 90.1% and a mean Heidke skill score, Hanssen‒Kuipers discriminant and Kuiper skill score over 0.8. All DM methods outperformed the Persistence model (assuming the persistence of the previous RWYCC based on manual observation), except for the Classification tree. The DM methods showed resiliency regarding the reliability of the input data, as they inherently used a poorly correlating input with less influence on the final result. 

The more precise the available information in the GRF is, the higher the increase in resilience, efficiency and safety in adverse weather situations (with rapidly changing conditions) can be expected. The quality of the information may be increased by qualitative and quantitative extensions of observation and calculation methods; both ways will be summarized below in more detail. 

The performance of DM methods grows as the available database of training data enlarges. Each extension of the validation period and the available input data brings potential improvement to DM models and increases the reliability of the results. The database can be extended temporally into the coming years and spatially by merging data from several airports. The latter, however, has to be done with care, as merging airports that are too dissimilar from climatic point of view can deteriorate the learning process. We plan to reexamine the results after each winter season and extend the number of testing sites, too.

A rule-based algorithm is another approach for the RWYCC calculation consisting of a physical model assessing all available input data with regards to the RCAM. In comparison with the DM methods, the performance of the rule-based algorithm turned out to be insufficient, due to the unreliability of the assessment of the contaminant type by runway built-in sensors having an undesirably large influence on the final output. Therefore, the rule-based algorithm should be modified so that the importance of sensor reported contaminant type will be suppressed in comparison to other inputs. On the other hand, the performance of the rule-based algorithm was not negatively affected by the uneven distribution of the reference measurements typical for rare phenomena. In conclusion, the most advantageous approach appears to be a combination of both the DM methods and the rule-based algorithm. DM methods would be utilized in common and frequent situations with a sufficient amount and distribution of data, and an upgraded rule-based algorithm (taking into account the revealed limitations) in rare conditions, when DM methods are limited by a lack of training data.

As a qualitative extension of the input database, we are also going to investigate the integration of new data sources, e.g., outputs of mobile runway surface condition sensors, surveillance data from an ADS-B receiver, on-line data of transmitted braking performance from landing aircrafts, processed imagery of ground-based cameras and cameras on aircraft. Such integrated validation exercise is planned for 2021 in Poland.

Furthermore, we plan to further apply our experiences with DM methods also to Lidar-based wind flow information to generate appropriate wind shear warnings. Finally, runway surface condition assessment and adverse wind condition detection together with a fog prediction model [4,5] would create a comprehensive warning system, targeting three of the most dangerous weather phenomena in aviation.

## Figures and Tables

**Table 1:** List of parameters used for the Runway Condition Code (RWYCC) computation. AWS stands for Automated Weather Station.

Parameter	Source of Data	Time Resolution
Sensor contaminant type [estimated code]	IRS31 Pro ^1^	1 minute
Observed contaminant type [according to ICAO Annex 15, Appendix 2 ^2^]	Previous SNOWTAM	Each manual inspection
Contaminant depth (water film height) [mm]	IRS31 Pro ^1^	1 minute
Contaminant depth [mm, reported values above 5 mm]	Previous SNOWTAM	Each manual inspection
Estimated BA [from 5 to 1]	Previous SNOWTAM	Each manual inspection
Runway surface temperature [°C]	IRS31 Pro ^1^	1 minute
Freezing point temperature [°C]	ARS31 Pro ^3^	1 minute
Air temperature in 2 m height [°C]	AWS	1 minute
Dew point temperature in 2 m height [°C]	AWS	1 minute
Precipitation Indicator [precipitation yes/no]	AWS	1 minute
Intensity of precipitation from disdrometer [mm/h]	AWS	1 minute
Type of precipitation from disdrometer [according to WMO table 4680 [10]]	AWS	5 minutes mean value each minute
Type of precipitation from METAR message [according to WMO table 4678 [10]]	METAR	30 minutes
Precipitation sum [mm]	AWS	10 minutes

^1^ Passive runway surface condition sensor; ^2^ SNOWTAM format according to International Civil Aviation Organization (ICAO) Annex 15 [9] before implementation of Amendment 39b introducing the Global Reporting Format (GRF); ^3^ Active runway surface condition sensor.

**Table 2:** Multi-category contingency table [29].

	Observed Categories					
**Forecast categories**	i,j	1	2	…	K	Total
	**1**	*n(F1, O1)*	*n(F1, O2)*	…	*n(F1, OK)*	*N(F1)*
	**2**	*n(F2, O1)*	*n(F2, O2)*	…	*n(F2, OK)*	*N(F2)*
	**…**	…	…	…	…	…
	**K**	*n(FK, O1)*	*n(FK, O2)*	…	*n(FK, OK)*	*N(FK)*
	**Total**	*N(O1)*	*N(O2)*	…	*N(OK)*	*N*

**Table 3:** Statistics for the scores of the Linear Discriminant Analysis (LDA) method.

	Mean	Sd	Var	Min	Max	Median	Skewness	Kurtosis
**ACC**	0.683	0.026	0.001	0.604	0.761	0.683	−0.120	2.920
**HSS**	0.297	0.051	0.003	0.146	0.437	0.296	−0.001	2.717
**HK**	0.256	0.047	0.002	0.121	0.399	0.255	0.076	2.743
**KSS**	0.406	0.072	0.005	0.201	0.674	0.406	0.098	2.899

**Table 4:** Statistics for the scores of the *K*-nearest Neighbors (KNN) method (*K* = 1).

	Mean	Sd	Var	Min	Max	Median	Skewness	Kurtosis
**ACC**	0.901	0.027	0.001	0.800	0.965	0.900	−0.320	2.901
**HSS**	0.809	0.051	0.003	0.628	0.932	0.813	−0.352	2.958
**HK**	0.806	0.058	0.003	0.585	0.959	0.812	−0.397	3.017
**KSS**	0.818	0.054	0.003	0.625	0.946	0.822	−0.320	2.806

**Table 5:** Statistics for the scores of the *K*-nearest Neighbors (KNN) method (*K* = 3).

	Mean	Sd	Var	Min	Max	Median	Skewness	Kurtosis
**ACC**	0.753	0.030	0.001	0.657	0.830	0.757	−0.132	2.750
**HSS**	0.511	0.058	0.003	0.312	0.673	0.512	−0.194	2.898
**HK**	0.492	0.063	0.004	0.277	0.689	0.495	−0.200	2.818
**KSS**	0.542	0.064	0.004	0.364	0.734	0.540	0.069	2.873

**Table 6:** Statistics for the scores of the Artificial Neural Network (ANN) method (training data set).

	Mean	Sd	Var	Min	Max	Median	Skewness	Kurtosis
**ACC**	0.708	0.046	0.002	0.067	0.836	0.705	−4.682	70.314
**HSS**	0.311	0.133	0.018	−0.139	0.676	0.310	0.093	2.575
**HK**	0.264	0.134	0.018	−0.235	0.652	0.248	0.333	2.711
**KSS**	0.548	0.112	0.012	−0.294	0.822	0.544	−0.955	10.062

**Table 7:** Statistics for the scores of the Artificial Neural Network (ANN) method (test data set).

	Mean	Sd	Var	Min	Max	Median	Skewness	Kurtosis
**ACC**	0.666	0.044	0.002	0.139	0.827	0.665	−2.825	35.872
**HSS**	0.219	0.112	0.012	−0.067	0.608	0.213	0.225	2.749
**HK**	0.187	0.109	0.012	−0.098	0.596	0.172	0.523	3.009
**KSS**	0.391	0.149	0.022	−0.256	0.827	0.385	−0.113	4.171

**Table 8:** Statistics for the scores of the Classification tree method.

	Mean	Sd	Var	Min	Max	Median	Skewness	Kurtosis
**ACC**	0.569	0.016	0.000	0.391	0.609	0.573	−3.781	39.544
**HSS**	0.103	0.029	0.001	−0.001	0.246	0.097	2.229	11.622
**HK**	0.089	0.027	0.001	−0.001	0.225	0.083	2.560	12.779
**KSS**	0.147	0.035	0.001	−0.001	0.295	0.140	0.976	7.173

**Figure A1:**
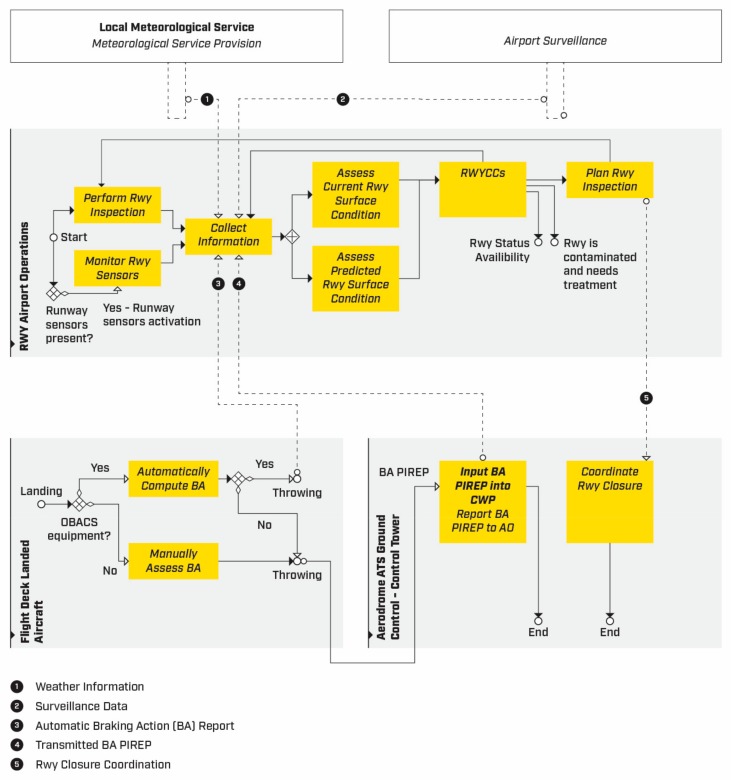
The new conceptual method for elaboration of the runway surface condition status based on the outputs of SESAR Pj03b-06 Solution.

**Figure A2:**
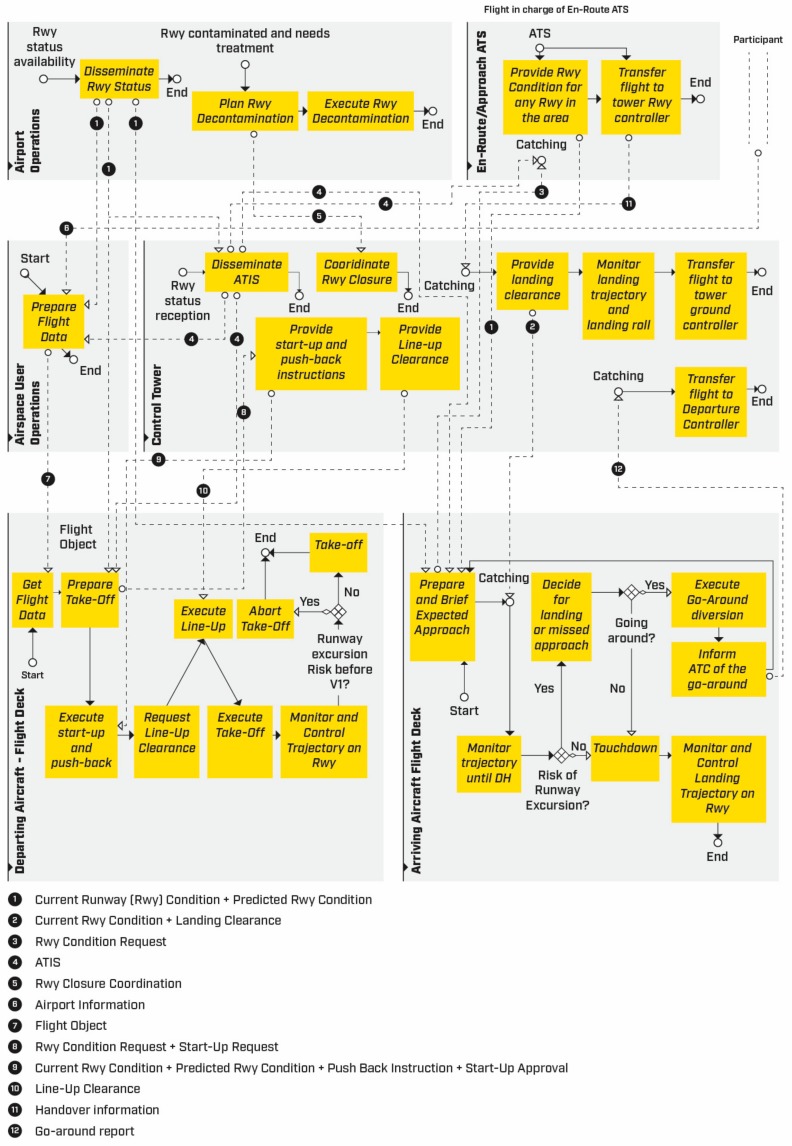
The new conceptual method for dissemination and utilization of the runway surface condition status based on the outputs of SESAR Pj03b-06 Solution.

**Figure A3:**
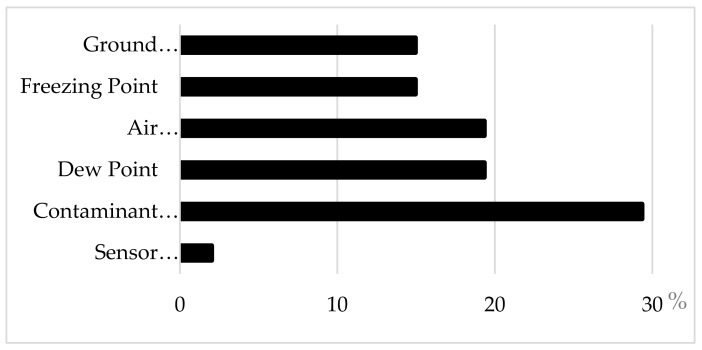
Contribution of weather parameters (in %) to the model developed by Aéroports de Paris within SESAR validation using data from Poprad–Tatry Airport.

## Appendix A

**Table A1:** Runway Condition Assessment Matrix (RCAM) [8].

Assessment Criteria		Downgrade Assessment Criteria	
**Runway Condition Code**	Runway Surface Description	Aeroplane Deceleration or Directional Control Observation	Pilot Report of Runway BA
6	DRY	-	-
5	FROST WET (The runway surface is covered by any visible dampness or water up to and including 3 mm depth Up to and including 3 mm depth: SLUSH DRY SNOW WET SNOW	Braking deceleration is normal for the wheel braking effort applied AND directional control is normal	GOOD
4	-15°C and Lower outside air temperature: COMPACTED SNOW	Braking deceleration OR directional control is between Good and Medium	GOOD TO MEDIUM
3	WET (“slippery wet” runway) DRY SNOW or WET SNOW (any depth) ON TOP OF COMPACTED SNOW More than 3 mm depth: DRY SNOW WET SNOW Higher than -15°C outside air temperature ^1^: COMPACTED SNOW	Braking deceleration is noticeably reduced for the wheel braking effort applied OR directional control is noticeably reduced	MEDIUM
2	More than 3 mm depth of water or slush: STANDING WATER SLUSH	Braking deceleration OR directional control is between Medium and Poor	MEDIUM TO POOR
1	ICE ^2^	Braking deceleration is significantly reduced for the wheel braking effort applied OR directional control is significantly reduced	POOR
0	WET ICE ^2^ WATER ON TOP OF COMPACTED SNOW ^2^ DRY SNOW or WET SNOW ON TOP OF ICE ^2^	Braking deceleration is minimal to non-existent for the wheel braking effort applied OR directional control is uncertain	LESS THAN POOR

^1^ Runway surface temperature should be used where available; ^2^ The AO may assign a higher RWYCC (but no higher than code 3) for each third of runway, provided the procedure in 1.1.3.15 [8] is followed.

## Appendix E

**Table A2:** List of abbreviations.

ACC	Accuracy
ANN	Artificial Neural Network
AO	Airport Operator
ATCO	Air Traffic Controller
ATM	Air Traffic Management
AWS	Automated Weather Station
BA	Braking Action
CWP	Controller Working Position
DM	Data mining
GRF	Global Reporting Format
HK	Hanssen‒Kuipers discriminant
HMI	Human‒Machine Interface
HSS	Heidke skill score
ICAO	International Civil Aviation Organization
KNN	*K*-nearest Neighbors
KSS	Kuiper skill score
LDA	Linear Discriminant Analysis
Max	Maximum
Min	Minimum
OBACS	On-board Braking Action Computation System
PIREP	Pilot Report
RCAM	Runway Condition Assessment Matrix
RCAMS	Runway Condition Awareness Management System
RCR	Runway Condition Report
Rwy	Runway
RWYCC	Runway Condition Code
Sd	Standard deviation
SESAR	Single Europe Sky Air Traffic Management Research
Var	Variability
WMO	World Meteorological Organization
